# *Plasmodium Vivax* causing acute kidney injury: A foe less addressed

**DOI:** 10.12669/pjms.316.8859

**Published:** 2015

**Authors:** Rubina Naqvi

**Affiliations:** 1 Prof. Dr. Rubina Naqvi Professor of Nephrology, Sindh Institute of Urology and Transplantation (SIUT), Karachi. Pakistan

**Keywords:** Acute Kidney Injury (AKI), RIFLE criteria, Malaria, *Plasmodium Vivax*

## Abstract

**Objective:**

To report patients developing acute kidney injury (AKI) after Vivax malaria.

**Methods:**

An observational cohort of patients identified as having acute kidney injury (AKI) after Plasmodium vivax infection. AKI was defined according to RIFLE criteria with sudden rise in creatinine or decline in urine output or both. All patients had normal size non obstructed kidneys on ultrasonography, with no previous co morbids. Malarial parasite Vivax was seen on blood peripheral film in all patients.

**Results:**

From January 1990 – December 2014, total 5623 patients with AKI were registered in our institute, of these 671 (11.93%) developed AKI in association with malarial infection, furthermore, Vivax was species in 109 patients. Average age of patients was 33.49±14.67 (range 8-78 years) with 66 male and 43 female. Oligo-anuria and vomiting were most common associated symptoms with fever. Renal replacement therapy required in 82 (75.22%) patients. Complete recovery was seen in 69 (63.30%), while 14 (12.84%) expired during acute phase of illness. Jaundice, thrombocytopenia, central nervous system involvement, mechanical ventilation requirement and hematuria were the factors significantly associated with high mortality.

**Conclusion:**

Malaria still causing significant morbidity and mortality in our part of world. Vivax malaria can present with hemolysis, thrombocytopenia and kidney failure in remarkable number of patients.

## INTRODUCTION

It was 1880 when a French physician, Charles Laveran, first time detected that causative organism for malaria is a sporozoan of the genus Plasmodium.^1^ The most common species of this genus which can affect human are *Plasmodium falciparum*, *P. malariae*, *P. Vivax*, and *P. ovale*. Infection is transmitted through bite of infected anopheles mosquito and may be life threatening in some. *Plasmodium falciparum*, being reported most deadly, whereas *P. Vivax*, historically used to be known as ‘benign tertian malaria’. *P. Vivax*, is most widely distributed human malaria, with global burden of 70-80 million new cases every year.^2^ Severe infection, as recognized with multi organ involvement, with *P. Vivax* has been reported by Kochar et al.^3^ It has been observed that in comparison to falciparum, vivax has lower pyrogenic threshold, more pronounced inflammatory response and higher cytokine production.^4^ The endothelial dysfunction leading to impaired microcirculation, a phenomenon of Falciparum malaria, has not been described in vivax malaria, but endothelial ‘stimulation’ has been reported at autopsy long time ago.^5^

AKI as result of *P. Vivax* infection, in 20% of malarial AKI has been reported from India.^6^ We have previously published our results from same institute on AKI resulting from malaria but in those times *P.Vivax* contributed to 2.4% of total malarial AKI.^7^ Here we aim to report our experience of markedly increasing renal involvement with *P.Vivax* infection.

## METHODS

This study is based on a cohort of 109 patients with AKI after infection with Vivax malaria. From a retrospective chart review of all patients admitted to the Sind institute of Urology and Transplantation, Karachi, Pakistan between January 1990 and December 2014. AKI was identified and staged according to RIFLE criteria.^8^ Only patients with recent past history of malaria were included in cohort. This duration ranged from one day to one month. Malaria was diagnosed on finding of parasite on Giemsa stain or modified Leishman stained blood peripheral film. Immunochromatographic technique was used to make diagnosis in patients who reached late after getting some treatment at peripheral hospital and reason for coming here was renal dysfunction. Only patients with no past medical history were included and all patients had normal size non obstructed kidneys on ultrasonography. Variables recorded for each patient on day of admission include age, history, duration of insult, oliguria, anuria, altered level of consciousness (ALOC), Hemoglobin, total leukocyte count, platelet count, prothrombin time, activated partial thromboplastin time, blood peripheral film, blood Urea, serum Creatinine, serum Sodium, serum Potassium, venous bicarb, serum lactate dehydrogenase, serum bilirubin, aspartate aminotransferase, alanine aminotransferase, gamma glutamyl transpeptidase, alkaline phosphatase, urine dipstick, urine microscopy, renal ultrasonography, parenteral fluids, renal replacement therapy, anti-malarial given before coming to our hospital and outcome. Renal biopsy was done in patients who revealed delay in recovery and ultrasonography was not supporting cortical necrosis or urine dipstick showed 3+ proteins.

### Ethical review

Institutional ethical review committee was approached to grant permission for publishing the data.

### Statistical methods

Quantitative variables reported as means ± SD and Qualitative as percentages. For univariate analysis of mortality risk factors student’s t-test applied, categorical variables summarized as frequencies and percentages and then analyzed by the Chi-square test or Fisher exact test where appropriate. Statistical analysis was done on SPSS version 20.0.

## RESULTS

Between Jan.1990 to Dec 2014, 671 patients developed AKI secondary to malaria of these 109(1.93% of total AKI) patients had Vivax malaria causing AKI, registered to our hospital. Average age of patients was 33.49± 14.67 years (range 8-78), with 66 male and 43 female.

Vomiting and oliguria were common associated symptoms with fever. Neurological involvement varies from drowsiness to frank coma. Diarrhea typically developed on second or third day of febrile illness. Three patients developed generalized tonic clonic convulsions during febrile illness. Eight patients required mechanical ventilation during acute phase of illness and only one among them survive. Hemorrhagic/purpuric rash was seen in 3 patients only (Table-I).

**Table-I:** Presenting symptoms and signs (n=109).

Symptoms / signs	No (%)
Fever	109 (100)
Oligo-anuria	76 (69.72)
Vomiting	72 (66.05)
Jaundice	42 (38.53)
Diarrhea	12 (11)
Altered level of consciousness	14 (12.84)
Convulsions	3(2.75)
Dyspnoea/Breathlessness	12 (11)
Hepatomegaly alone	14 (12.84)
Spleenomegaly alone	4 (3.66)
Both liver and spleen enlarged	4 (3.66)
Hemorrhagic /Purpuric rash	3 (2.75)

Most of our patients had anemia, thrombocytopenia, and advanced uremia, 13% had high serum potassium levels and 24% had low sodium on presentation. International normalized ratio for coagulation was found deranged in 15 (13.7%) patients. Urinalysis was available from 75 patients, on dipstick Proteinuria of 1+ to 3+ was found in 43 of these and microscopic hematuria in 61 patients (Table-II).

**Table-II:** Laboratory Findings (n=109).

Parameters	Values
Haemoglobin mean ± SD in g/dl	9.94+3.24 (range 2.6-19.3)
Platelet count mean ± SD	147.88 +146.39 (range 2,000-777,000)
Blood Urea mean ± SD mg/l	238.25 +95.49 (range 62-543)
Serum Creatinine mean ± SD mg/l	10.01 +4.44 (range 2.6 -22.98)
Deranged INR (%)	15 (13.7)
Hyperkalemia K>5.5 meq/l(%)	14 (12.84)
Hyponatremia Na <130 meq/l(%)	27 (24.77)
Microscopic hematuria (%)	61(81.33)*urinalysis available for 75 patients
Proteinuria 1-2+ on dipstick (%)	43 (57.33))*urinalysis available for 75 patients

Renal biopsy was carried out in 15 patients, findings were acute tubular necrosis in 6, cortical necrosis in 4, tubulointerstitial nephritis in 2 and 2 had crescentic glomerulonephritis. Among 4 who had cortical necrosis 3 partially recovered and one developed end stage kidney failure. Overall 3 patients progressed to end stage, in two among them ultrasonography was suggestive of acute cortical necrosis, described by hypo echoic rim under capsule, thus they were not subjected to renal biopsy. Renal replacement therapy was required on arrival in 82 (75.22%) cases, it was hemodialysis in all.

Complete recovery seen in 69 (63.30%), while 14 (12.84%) expired during acute phase of illness, partial recovery was found in 22 (20.18%) and 3 patients developed end stage renal failure.

Jaundice, thrombocytopenia, central nervous system involvement, mechanical ventilatory support requirement and microscopic hematuria were the factors associated with high mortality with p value of < 0.05 (Table-III).

**Table-III:** Indicators of mortality and their significance.

Parameters	Survived (n=95)	Expired (n=14)	P value
Age mean ±SD in years	33.44 ±14.22	33.86±18.0	0.922
Days of insult mean±SD	9.71±6.64	10 ± 7.41	0.019
Excessive vomiting	65	7	0.146
Oligo-anuria	66	10	0.287
ALOC	7	7	<0.001
Jaundice	33	9	0.03
Thrombocytopenia	59	11	0.02
Deranged coagulation	12	3	0.650
Hyponatremia (Na ≤130meq/l)	22	5	0.490
Hyperkalemia (K ≥5.5meq/l)	11	3	0.288
Proteinuria 1-2+ on dipstick	40	3	0.218
Microscopic hematuria	57	4	0.008
Mech. Ventilation required (no. of patients)	1	7	<0.001

## DISCUSSION

Acute kidney injury as serious complication of P. Falciparum has been widely reported in literature^9^,^10^, but P. Vivax causing AKI is less addressed issue and been reported over last one and half decade, however, mostly published are small case series.^10^ In our own experience we published malarial AKI a series of 124 cases where only 3 had Vivax malaria.^7^

A review on pathophysiology of Vivax malaria published by Anstey et al in 2009, highlights different mechanisms of severity of disease and organ involvement.^4^

Parkash et al. have published a series of 19 cases where AKI resulted from Vivax malaria, have observed encephalopathy and jaundice in 58% cases^6^, whereas in present cohort based on 109 cases we have observed 13 and 39% respectively. But both of these factors found in significant relation with mortality rate in our studied population.

Symptomatology from Vivax malarial AKI reported from different centers share many symptoms in common. Decline in urine output has been reported from 47%^6^ to 84%^11^ in present study we found oligo-anuria in 69.72% of patients. Cerebral involvement, which varies in reports from altered sensorium to coma, has been reported from 36%^11^ to 58%^6^ we have seen in 12.84% in present study which is far less than previous reports but we had larger number of patients then those reported in past (11 and 19 patients with Vivax malaria).

Hyponatremia is a typical biochemical finding, being reported in up to 15.8% of cases with severe Vivax malaria^6^, we found in 24.77% of our patients. Hyperkalemia is striking feature, and often fatal with its cardiac arrhythmias complication. It is attributed to hemolysis and acidosis, particularly in setting of renal failure. In our studied population 13% suffered from hyperkalemia but its contribution to mortality was not significant. Coagulopathy from previous studies been reported very high by Parkash et al. (in 42%)^6^ whereas in present study it was 13.7%. Collective incidence of diarrhea in association with malarial AKI, from different series has been reported 6%^1^, in present study we found it 11%. Seizures has been reported in severe Vivax malaria cases specially in those who had cerebral involvement^3^ in present series we see 3 patients experiencing convulsions and all three had cerebral involvement. Acidosis is another factor which may contribute to clouding sensorium and seizures. Rash in our patients particularly found in thrombocytopenic ones, as average patients in this cohort had low platelet count, minimal were 2000. Thrombocytopenia in the majority of cases is part of a disseminated intravascular coagulation initiated by the gross rheological abnormality in severe malaria.

The whole spectrum of morphological changes on renal histology has been reported in malarial nephropathy, acute tubular necrosis resulting from hemodynamic changes, acute interstitial nephritis refractory to tubular injury or resulting from acute inflammatory response, then glomerular changes as part of acute post infectious process or indicating immune mediated changes. These are well described in previously published review articles.^1^,^12^ In our present cohort we were able to find all verities of histological changes.

Three quarters of our studied population required renal replacement on arrival, reason being, this is a tertiary care hospital and patients are referred here from all over the country and majority of patients are referred only when they are in need of renal replacement, by their primary treating physicians.

Infection with plasmodium Vivax which was thought to be more benign in course is no more a reality and over last about one and half decade we see this species causing whole spectrum of pathogenesis quite similar to Falciparum. In literature; hemolysis, DIC, hypotension and hyperbilirubinemia from Vivax malaria has been reported from India, in two case series of 19 and 40 cases.^3^,^6^

Mortality in cases with vivax malaria and AKI has been reported from 11 to 16%.^3^,^6^,^13^ Present study shows higher mortality than other reported series but slight decrease from past experience from same center.^7^ Previously we have reported cerebral involvement and coagulopathy as poor prognostic factor in malarial AKI,^7^,^14^ others have also reported CNS involvement, DIC, sepsis, jaundice, anemia, hypotension and severe decline in urine output as poor prognostic factors.^10^,^15^ In present study which is limited to Vivax malaria cases thrombocytopenia, nervous system involvement, mechanical ventilatory requirement and microscopic hematuria were found in significant association with mortality.

Some of finding in current study may be biased for the reason that majority of patients arrive in advance stage of uremia and some findings may be contributed with uremia rather then vivax infection by itself.

## CONCLUSION

Vivax is more than “benign” tertiary malaria and can give rise to whole spectrum of pathological changes leading to high morbidity and mortality. There is need of reporting such morbidities and increasing awareness in medical community and society at large.
